# New Appliances and Things Medical

**Published:** 1897-10-23

**Authors:** 


### NEW APPLIANCES AND THINGS MEDICAL.

[We shall be glad to receive, at our Office, 28 & 29, Southampton Street, Strand, London, W.C., from the manufacturers, specimens of all new preparations and appliances which may be brought out from time to time.]

### VIN DESILES.

(Sevin, Rue du Louvres, Paris.)

This is a new restorative cordial, which appears to have obtained some popularity in France. Containing as it does a certain proportion of quinine, coca, kola, phosphate of lime, and other nerve tonics and stimulants, it is not astonishing that many persons suffering from nervous exhaustion and debilitating conditions should have derived considerable benefit from its administration. The dose is stated to be two or three wine glasses per diem, after or before meals. The want of exact information as regards chemical composition and therapeutic action will probably prevent this cordial from being largely prescribed by the profession. The public, however, who abide by empirical experience, will doubtless receive with favour its introduction to the English market.

### CONDENSED MILK (VIKING BRAND), UNSWEETENED.

(Norwegian Milk Condensing Company, Limited).
(Agents: Anderson and Coltman, 5, Philpot Lane, E.C.)

A really good unsweetened condensed milk is a desideratum from two points of view. Firstly, it can be used for tea, coffee, and cocoa by those who dislike or cannot digest excess of sugar; and, secondly, for the use of infants, the absence of added cane sugar is of great importance. Analysis of the Viking brand shows that the three chief constituents, namely, fat, albumin, and sugar, stand in practically the same proportion to one another that they do in fresh milk of good quality. There is perhaps a slight deficiency of albumin as compared with fresh milk, but for the feeding of children this is an advantage rather than otherwise. We cannot detect the presence of any objectionable added matter for the purposes of preservation, but according to the analysis supplied by the company, the mineral matter is slightly in excess as compared with ordinary milk. That is to say, taking the ratio between the mineral matter and the guaranteed quantity of fat, casein, albumin, and milk sugar in the Viking brand, it stands as 2·12 to 35·66. Roughly speaking, in ordinary cow's milk the ratio is ·7 to 12·25. This difference in the ratio is probably due to the slight deficiency of albumin. It cannot, however, affect the value of the milk from a dietetic point of view. The full quantity of cream present in the Viking brand and the absence of added sugar puts it in a prominent rank among canned milks, and if only those people who feed their children exclusively on condensed milk were to employ this brand instead of many others, which are sadly deficient in cream and contain a great excess of sugar, there would be fewer badly-nourished and sickly children.

